# The Sexual Development, Sexual Health, Sexual Experiences, and Sexual Knowledge of Forensic Mental Health Patients: A Research Design and Methodology Protocol

**DOI:** 10.3389/fpsyt.2021.651839

**Published:** 2021-06-04

**Authors:** Elnike Brand, Angela Ratsch, Edward Heffernan

**Affiliations:** ^1^Faculty of Medicine, University of Queensland, Brisbane, QLD, Australia; ^2^Wide Bay Hospital and Health Service, Research Services, Hervey Bay Hospital, Hervey Bay, QLD, Australia

**Keywords:** mental health, psychiatry, forensic, sexuality, sexual health, serious mental illness, survey questionnaire

## Abstract

**Introduction:** There is substantial evidence that severe mental illness (SMI) can have significant impacts on general development, knowledge acquisition, and quality of life including sexual function. However, whilst the sexual development, sexual health, sexual experiences and sexual knowledge of the broader Australian community and the Australian prisoner population have been well-described, these concepts have been less explored in people with a SMI. In particular, there is an absence of research around these topics involving people who are subject to a treatment order (aka a Forensic Order) under the relevant jurisdictional Mental Health Act.

**Methods and Analysis:** People currently under the treatment requirements of a Queensland Forensic Order will be invited to participate in this descriptive, mixed-method study. The study will be conducted in three phases. The first two phases will involve 50 participants (100 face-to-face quantitative interviews) with the aim of mapping sexual development, sexual health, sexual experiences, and sexual knowledge. The third phase will involve qualitative semi-structured interviews with a purposely enrolled, informant-rich cohort identified through the quantitative surveys until saturation is reached. Quantitative data will be analyzed using descriptive and comparative statistics. Qualitative data will be analyzed by content analysis of the major themes.

**Ethics and dissemination:** The project has ethics approval from a Queensland Health Human Research Ethics Committee. Results will be reported to participants and other stakeholders at seminars and conferences and through peer-reviewed publications.

**Strengths and Limitations of this Study:** This is a mixed-method study which engages with participants by using face-to-face interviews. The study is conducted in three phases which sub-divide the research into the following areas: (1) demographics, general health, and sexual health, (2) sexual knowledge and experiences, and (3) sexual knowledge gaps. This study uses electronic data capture to efficiently record and analyse participant responses. This study captures self-reported data and uses non-probability sampling from a population who have been pre-selected through an arms-length approach—both these processes elevate the risk of bias.

## Introduction

At a global level, the World Health Organization (WHO) recognizes the importance of sexual health and sexual functioning to recovery and health, and defines sexual health as, “a state of physical, emotional, mental, and social well-being in relation to sexuality; it is not merely the absence of disease, dysfunction or infirmity. Sexual health requires a positive and respectful approach to sexuality and sexual relationships, as well as the possibility of having pleasurable and safe sexual experiences, free of coercion, discrimination, and violence. For sexual health to be attained and maintained, the sexual rights of all persons must be respected, protected and fulfilled” (1).

Whilst this generalized affirmation is supportive and encouraging of the normalization of sexual health into the concept of overall health, nonetheless, there remains a significant gap in the literature to inform the understanding of the sexual development, sexual health, sexual experiences, and sexual knowledge (hereafter termed sexuality and sexual health) of people with a SMI (i.e., people with a diagnosis of non-organic psychosis, a duration of treatment of two years or more, and significant dysfunction).

### Mental Health and Sexual Health

Mental health and sexual health are bi-directional constructs—a person's mental health affects their sexual health, and a person's sexual health affects their mental health (2). However, it would seem that with the diagnosis of a SMI, the person becomes a “patient,” and with that transition, invariably their health needs are prioritized to their mental health requirements. This prioritization is to the extent that the concept of their sexuality and sexual health, as part of their holistic health care is diminished, that is, their functioning as a whole being is obviated (3).

Literature from several perspectives informs the nature, approach and conduct of this study. The first is the Australian Study of Health and Relationships 2 (4) which was conducted from 2012–2013 and surveyed the general Australian population (*n* = 19,307). The general population data was extended by Butler et al. (5) who investigated the sexual health and sexual behavior of Australian prisoners. McMillan et al. (3) followed those reports by focusing on particular aspects of the sexual functioning and experiences in young people affected by SMI.

Additional literature shows that some clinicians have a knowledge of sexology, and many more have a knowledge of mental health, the gap however is at the intersection of sexology and mental health. This gap is multifaceted and complex. De Jager et al. (6) points out that mental health clinicians often lack awareness of the sexual health and sexuality of patients diagnosed with a SMI. From an Australian clinician perspective, Urry (7) and Quinn (8) both explored the difficulty discussing sexuality with patients. This clinician-patient hiatus is extended when clinicians avoid or negate sexuality and sexual health as an important health assessment topic (9). Knegtering and Bruggeman (10) contend that this lack of awareness, avoidance, or negation, produces a lack of clinical awareness and recognition of the impact that many mental health conditions and treatments can potentially have on sexual functioning.

Further compounding and widening the sexology-mental health-clinician gap, Quinn et al. (11), Evans (12), and Brand et al. (13) emphasis the lack of, and barriers to positive sexual experiences for patients with mental illness and show that patients' are reluctant to self-report sexual dysfunction to clinicians. This reluctance is contrary to the anecdotal evidence presented by Baggaley (14) which suggests that sexual dysfunction has a considerable impact on quality of life (15) and adherence to antipsychotic medications (16). Montejo (17) also identify that SMI symptoms and medications which affect sexual life, including impaired desire, arousal, or sexual satisfaction, are often not addressed, or where attempts by clinicians to address the concerns are made in a recovery orientated model of care, organizational governance issues are created (18).

These gaps and impediments around clinician-patient knowledge and communication impact the clinical interaction and can result in a failure to address a substantial problem for the patient, and potentially complicate and delay the patient's overall recovery and rehabilitation from SMI (19). This is even more prominent in the field of forensic mental health, were patient's recovery focused treatment and rehabilitation spans decades.

### Research Question, Aim, and Objective

SMI are by definition, pervasive and impact significantly on functionality (20). SMI impacts all aspects of development and quality of life, including sexual function. Conversely, for people whose SMI affects their sexuality and sexual health there is an absence of: (1) evidenced-based guidelines or approaches; and (2) support structures to engage in socially appropriate experiences to achieve a healthy sexual life. These two points are particularly important given the young ages at which SMI tends to develop, and their often life-time presence.

Within the category of “extremely unwell patients with a SMI” are those who are under the treatment requirements of a Forensic Order. These patients have been charged by police with a serious criminal offense, however, have been found to be of “unsound mind (insane)” (21) at the time of the alleged offense, or unfit for trial (22). Accordingly, the research question is, “What is the sexual development, health, experiences and knowledge of people with SMI being treated under the requirements of a Forensic Order?”

## Methods and Analysis

### Study Design

Patient and Public Involvement: Patients, as research participants, are prospectively recruited to this study. The research question, design and data collection tools have been developed in consultation with a key community stakeholder and with local and state-wide mental health clinicians and academics.

Human sexuality is complex and is inclusive of a number of phenomena, including sexual behavior, desire, pleasure, sexual identity and orientation. SMI is equally as complex, thus adequately capturing the diversity of individuals' tangible sexual experiences against a background of major SMI is challenging and this is demonstrated by the limited volume of quantitative and qualitative evidence-based literature at the interface of sexology and mental health. Accordingly, for this descriptive study, a mixed-method design will be used with prospectively enrolled participants to address the research question and with the aim of acquiring data to inform further research. The research aims to gather the data in such a manner that comparison with existing literature can be undertaken, and additionally, if deficits are identified, evidence-based intervention programs could be developed to support this population to experience healthy sexual lives.

### Methodology

This study will be conducted in three consecutive phases. Phases 1 and 2 will comprise of two quantitative studies and will utilize validated or survey questions from published research. Phase 3 will use a qualitative approach and be informed by the findings from Phase 1 and 2. All three data collection phases will be conducted using face-to-face interviews by an experienced forensic psychiatrist and sexologist, who has worked clinically in the field of forensic psychiatry for over 20 years.

### Population, Sampling, Recruitment, and Consent

The participant cohort will be community-based patients being treated under the requirements of a Forensic Order (Queensland). Patients being treated under the requirements of a Forensic Order have a major SMI diagnosis that has been confirmed by a number of psychiatrists and mental health services, and the diagnosis has been accepted by a court of law. These people have specific legal requirements to regularly attend mental health services. Accordingly, a very structured and supportive clinical network is responsible for providing appropriate care and managing treatment to ensure and enable these patients to engage in recovery and rehabilitation. This team includes an authorized doctor and a Forensic Liaison Officer (FLO). FLOs are registered clinicians including nurses, psychologists, and occupational therapists and have specialized training in mental health. The FLO leads the multidisciplinary mental health team and coordinates the holistic care provided to Forensic Order patients. They advocate for the patient and are the conduit between the patient and the multidisciplinary team.

### Sample

All people treated under the requirements of a Forensic Order in three Queensland Hospital and Health Services (HHSs) and who meet the inclusion criteria (Table 1) and are considered well-enough to participant will be offered enrolment. Potential participants will be invited to participate in any or all phases of the research. The index offense for which the Forensic Order was made will not be taken into account when recruiting the population. Potential participants who are judged by the FLO to be too unwell to participate (by virtue of mental or physical health problems) or who cannot consent, for example, those who have a cognitive impairment, will be excluded. All participants with an intellectual disability (Full scale IQ < 70) will be excluded from the study. People who are not proficient in the spoken English language will be included and the services of a professional interpreter will be arranged.

**Table 1:** Inclusion and exclusion criteria.

**Inclusion criteria**	**Exclusion criteria**
≥ 18 years Receiving treatment under the requirements of a Forensic Order Capacity to consent and participate Community-based	IQ < 70 Vulnerable people – recent exposure or disclosure of sexual abuse or assault Hospitalized for SMI

Within the study area, the current population of potential participants is *n* = 215, however a portion of these will not meet the eligibility criteria. For this descriptive study, 50 completed Phase 1 and 50 completed Phase 2 surveys (~20% of the population) is considered sufficient to address the research questions and provide information for future studies. Given the potential for drop-outs prior to, during or throughout the phases of the study, possible recruitment could be up to 60 participants, however, as there is a small financial incentive to complete the survey, the FLO and treatment teams advise that the drop-out once enrolled will be minimal.

### Recruitment

A “hands off” approach to recruitment will be employed. Potential participants will be identified and informed by the FLO of the research project and will indicate their agreement to consider participation by completing an *Approval to Provide Contact Information to Researcher* form. Only these potential participants will be assessed in a desk-top review by the treating clinician team and interviewer for suitability to participate and their names will be placed on the “deemed eligible list.” Table 2 details the steps in the identification and recruitment process.

**Table 2:** Identification, recruitment and consent processes.

**Steps**	**Process**
Step 1	The interviewer will liaise with the FLO across the three Hospital and Health Services (HHS) sites to discuss the project requirements. Research information sessions will be conducted with the FLOs to outline research project and details, clarify inclusion and exclusion criteria and discuss any concerns or issues.
Step 2	The initial screening process with no participant involvement will occur where the FLO will identify potential exclusion participants (e.g., recent exposure or disclosure of sexual abuse, IQ < 70 and hospitalized patients).
Step 3	The FLO will pass the *Research Information Sheet* onto the identified potential participants who will have at least 7-days to read the information and ask questions of the FLO. The FLO can contact the interviewer for any clarification in response to the questions.
Step 4	Potential participants who wish to be included in the research return the *Approval to provide contact information to researchers* form to the FLO. The FLO, treatment team and researcher will conduct a desktop review of the potential participant's current health status, and if appropriate to participate, will enter their names onto the ‘deemed eligible to participate’ list in the chronological order that the contact form was returned.
Step 5	Potential participants will be identified consecutively from the deemed eligible list, and the interviewer will arrange contact where both the FLO and the potential participant can be present for the consent.
Step 6	The interviewer will discuss the research with the potential participant. At this time, any additional questions can be identified and clarified with the interviewer.
Step 7	If the potential participant agrees to participate, the formal *Participant information and consent* procedures, including the witness by the FLO, will be obtained. It will be explained to participants that they can choose to withdraw consent at any time during the interview and that in addition, the interviewer can decide to discontinue the interview process at any time.
Step 8	The interviewer will conduct an informal assessment with the focus on the participants' capacity to consent and mental state (1) during the consent process, (2) prior to commencing the interviews, and (3) during and after the interviews. The participants will be encouraged to discuss any concerns, distress or discomfort with the interviewer at any time.
Step 9	At the completion of the survey, each participant will be explicitly asked as part of the research questions ‘How embarrassing did you find the questionnaire?’ and will be debriefed by the FLO. The debriefing will be a casual conversation to provide the participant with the opportunity to discuss any distress and discomfort and to enable the FLO to assess for any signs of distress.
Step 10	Should the participant experience significant distress during the interview, with or without other emerging symptoms of acute major SMI, the local FLO will be informed. The local FLO will have the opportunity to escalate any concerns to the local mental health treatment team. The interviewer will, in case of major concerns regarding a participant's mental state or risks, arrange for an emergency assessment by the acute care mental health teams in the area following the HHS pathways for an emergency assessment of people suspected of experiencing a relapse of SMI and increased risks to self and others. The FLO and treating teams will be informed if this step were deemed necessary.
Step 11	An adverse research event will be lodged and discussed with the researcher and clinical team.

The participants on the “deemed eligible list” will be consequently offered recruitment. Phase 1 will recruit and interview participants until 50 interviews are completed. At the completion of the interview, the participant will be offered enrolment into Phase 2. If they decline, additional recruitment for Phase 2 will be conducted until 50 interviews are completed. Following the Phase 1 and 2 interviews, information-rich participants (23) will be identified for the Phase 3 qualitative interviews and enrolments will continue until saturation is reached. Information-rich participants are those who are identified as wanting to provide in-depth understanding and have the vocabulary to support their sharing of knowledge.

Participants in Phase 1 will be remunerated for their time (1-h interview) with a $50 grocery voucher, those in Phase 2 with a $20 grocery voucher (30-min interview) and those in Phase 3 with a $50 grocery voucher (1-h interview). The intention is to complete the three phases within a 6-month period (see Table 3).

**Table 3:** Study phases and data collection.

**Study phase**	**Approach**	**Sample size**	**Data collection technique**	**Survey focus**	**Survey duration**
Phase 1	Quantitative	*n* = 50	Electronic questionnaire	Sexual knowledge	30 min
Phase 2	Quantitative	*n* = 50	Electronic questionnaire	Sexual development, identity, experiences, sexual health, alternative and non coital sexual behavior, sexual difficulties, and attitudes	60 min
Phase 3	Qualitative	*n* = 15	Interview with audio recording	Exploring in detail aspects of sexual development, sexual health, sexual experiences and knowledge	30 min

### Consent

Given that mental state can vary over a time period, the participants will be screened throughout each recruitment phase, including during and after the interviews for any signs and symptoms of emerging SMI, which might potentially influence their capacity to consent or participate in this research project. The interviewer will take into account this potentially vulnerable populations' nature of their condition, their medication and treatment needs, the level of distress that they will be able to tolerate, and the complexity of the questions asked before, during and after consent, and at each interview.

The interviewer is skilled and equipped to assess capacity to consent and the mental state of the participant both formally and informally, prior to and during the interview, based on their presentation and answers to questions. The interviewer will assess patient distress both subjectively and objectively. Given that the data collection will be conducted as a face-to-face interview, this assessment of distress is a standard component of clinical practice for the interviewer.

### Survey Contents and Data Collection

Interview schedules have been developed to gain participant responses across a wide range of domains in one-off face-to-face interviews. The team is interested in capturing the participant's initial responses to the survey questions and to prevent the risk of a second interview process and recall bias. The participants will not have the opportunity to review or edit their responses after the completion of the interviews.

All questions have been designed using simple language, are easy to understand, and can mostly be responded to with monosyllabic answers. Participants will also have the opportunity to have as many breaks (and for as long as they require) during the interview process, and to have a support person present for the duration of the interview if they wish. Although sexual abuse is a criterion of exclusion, it cannot be ruled out that patients are stimulated by the interview questions to talk about abuse for the first time. In addition, the questions have the potential to unmask underlying psychological distress. The interviewer is a forensic mental health trained clinician who has extensive experience working clinically in the field of forensic psychiatry. Should the participant disclose sexual abuse, the interviewer will be able to gently guide and support the participant to the focus of the questions, and should any participant become distressed, the interviewer will determine if cessation of the interview is appropriate. In all instances of disclosed sexual abuse and/or distress, the local FLO will be informed at the completion of the interview; this will enable the concern to be a focus of treatment and management for the regular Mental Health Treatment Teams.

Debriefing techniques will be used if the participant becomes distressed during or at the end of the interview. If the distress impacts on the mental state of a participant, or if any risks to self and other are identified as a result of disclosure of such information, an escalation will be enacted. The risk of distress will be minimized by providing as much information about the study in both verbal and written format prior to the commencement of the interviews in order for participants to make a fully informed choice around participation.

The interview schedules have been converted to an electronic format for ease of data collection and analysis. The interviewer will read out the questions and will reword each using language more fully understood by the participant if required. The interviewer will enter the responses directly into the electronic questionnaire and these responses will automatically populate a password secured, cloud stored, electronic database. Should there be an internet failure, a hard copy of the tool will be used.

The Phase 1 survey (Supplementary Material 1) will take ~30-min to complete. The questionnaire will utilize an electronic format of the well-known and validated General Sexual Knowledge Questionnaire (24) (GCKQ). The decision to use the GSKQ was based on the researcher's knowledge around the participant population's general lower level of education, and literacy and vocabulary skills. The GSKQ has been used successfully in a range of populations including general populations, sexually offending and non-offending groups, and those with and without intellectual disadvantage (24). The questionnaire consists of a series of short sentences, and a male and female image to be labeled. There is good evidence in the literature supporting the questionnaire's reliability and in addition, data sets (24) exist to provide comparative analysis for this research.

The Phase 2 survey (Supplementary Material 2) will take ~1-h to complete and is based on the survey questions used in the Sex in Australia 2 (25) and Sexual Behavior and Sexual Health of Australian Prisoners study (5), as well as four tools commonly used in mental health research: (1) the Sexual Dysfunction Questionnaire (SDQ); (2) the Antipsychotic and Sexual Functioning Questionnaire (ASFQ); (3) the Brief Psychiatric Rating Scale (BPRS;) and 4) the Medication Adherence Scale (MARS). The utilization of these data collection tools will enable the external validation of the research findings. The survey consists of many multiple-choice questions, however, the questions are brief and the responses required are equally brief. The questions cover a broad range of categories including: demographic details; general health; alcohol and substance use; primary, secondary, vocational and tertiary education; attitude toward mental health workers; quality of life; sexual identity; sexual development; physical development; first sexual experiences; emotional experiences with the last sexual contact; recent and past sexual partners; masturbation; alternative and non-coital sexual behavior; sex and the internet; sex work; sexual coercion; domestic violence; sexual difficulties; contraception; sexually transmitted infections; body modification; sexual education and knowledge; and sexual attitudes.

Phase 3 will consist of a semi-structured interview. The questions for this phase will be developed from the Phase 1 and 2 findings. The intent of this phase is to explore in detail aspects of sexual development, sexual health, sexual experiences, and knowledge identified as being influential, significant or principal from the earlier phases in order to fully address the research question. It is expected that the interview will take 1-h and will be audio recorded and transcribed prior to analysis.

### Data Processing

Data will be imported from the spreadsheet into SPSS® version 25.0 (IBM® SPSS® Statistics 25) for Windows for analysis. Validation of the data accuracy following transfer will be conducted by the researcher and a statistician. They will compare all computed values on the spreadsheets with all computed values in SPSS®. Missing data and outliers will be reported but will be excluded from analysis. Results will be reported with the level of significance at alpha.05 and accompanied by 95% confidence intervals (95% CI). The data will be analyzed using descriptive statistics to consider the research findings. Means ± SD or proportions will be reported where applicable. Data will be grouped into the major topics (sexual development, sexual health, sexual experience, and sexual knowledge) and then be examined by strata (e.g., age, education level, age at first sexual encounter, marital status, children).

Content analysis of the qualitative data from Phase 3 will be conducted using NVIVO® with codes generated, and the major recurring themes, patterns and relationship examined. All data will be transcribed literally. Themes of analysis will be arrived at by classifying the content into themes which could be a word, a phrase or sentence. The themes will be based on the objectives of the study. Sub-categories and a coding scheme for the analysis will then be developed. These will be derived from the primary data, theories on similar topics, and empirical studies. The content analysis will be based on a deductive approach and the interpretations will be linked to the existing theories to draw inferences. The coding scheme will be pre-tested to ensure consistency in coding by the research team members. Following testing, the coding process will be applied to the data and the whole data set will be checked for validly and reliability. Interferences will then be drawn based on the codes and categories. These will be explored to identify the relationships and patterns in order to present the analysis. The results will be presented under each theme with each conclusion supported by secondary data and quotes from the developed codes. Based on the analysis, graphical representations, matrices, or conceptual frameworks will be produced to assist the basis for the interpretations.

### Potential Risks

For the participant, potential risks include psychological harm such as feelings of worthlessness, distress, guilt, anger, or fear related to disclosure of sensitive or embarrassing personal information and legal harms including discovery and prosecution of criminal conduct. Participants will be informed that if serious previously unreported criminal conduct by themselves is raised, the interviewer is obliged to report it.

### Mitigation Strategy

Given that mental illness can vary over time, the participants will be screened throughout the recruitment phase and during and after the interviews for any signs and symptoms of emerging mental illness that might potentially influence their capacity to consent or participate in this research project. Also, as part of the research questions, the participants are asked explicitly how distressing/uncomfortable they found the questionnaire. At the completion of the interview, the participant will be debriefed by the FLO and be given the opportunity to discuss any distress and discomfort.

### Ethics and Dissemination

Ethical and scientific review has been obtained for the three phases of the study from the Human Research Ethics Committees (HREC) at the Royal Brisbane and Women's Hospital (HREC/17/QRBW/674, HREC/18/QRBW/56 and HREC/19/QRBW/53345) and the University of Queensland.

### Dissemination of Findings

Findings will be reported using the STROBE guidelines for observational studies (26). Participants and healthcare consumers will be informed of the project results through publication on publicly accessible websites, media, and local newsletters. Additionally, organization newsletters will contain details of the research findings and links to the journal articles. Findings will be presented at Australian and international conferences and seminars.

## Conclusion

### Limitations and Strengths

This is a small, descriptive, self-reported survey study involving a homogenous group of participants who have a SMI and who are under prescribed treatment and legal limitations. The nature of such a study can lead to the misreporting of actual experiences and behaviors. In addition, the enrolled participants' responses may vary from non-participant Forensic Order patients, however this comparative measure is unknown given this is the first study of its kind conducted within this population. The interviews are conducted as a one-off event, and whilst the participants are informed of the nature of the questions in the week(s) prior to the interview, the participants have had no opportunity to read the questions beforehand, nor will they be able to add to their responses after completion of the interview. This approach may limit the comprehensiveness of participant's responses and impact on the data collection and correspondingly, the analysis and findings. The study has several strengths. The first is in providing this population an opportunity to participate in research that focuses on improving knowledge and understanding of an aspect of their unique condition, and ultimately, improving their quality of life.

Focusing on the sexual development, sexual health, sexual experiences, and sexual knowledge in this specific research population is important. The findings will improve the understanding of sexuality and sexual health and in addition, this data will be used to inform interventions designed to address unmet sexual health or sexual knowledge needs in this group. Secondly, the introduction identified gaps in the sexual health and sexuality assessment of patients with a SMI, possibly associated with clinician's knowledge, attitudes, and behavior around these topics. Two derivate projects have thus far risen from this research study. The first is examining the attitude and knowledge of mental health staff regarding their patient's sexual health and well-being. The second study is exploring the inclusion of sexual history taking and documentation as part of mental health reviews.

In conclusion, this study aligns with the recovery principles in mental health. From the perspective of the individual with SMI, recovery means gaining and retaining hope, understanding of one's abilities and disabilities, engagement in an active life, personal autonomy, and social identity. Importantly, it is also about finding meaning and purpose in life, and a positive sense of self, which includes one's sexual self.

## Ethics Statement

The studies involving human participants were reviewed and approved by Royal Brisbane and women's hospital human research ethics committee. The patients/participants provided their written informed consent to participate in this study.

## Author Contributions

EB, AR, and EH: methodology and writing—review and editing. EB and AR: data collection/assembly and resources. EB: conceptualization, writing—original draft preparation, and funding acquisition. All authors have approved the submission and contents of this manuscript.

## Conflict of Interest

The authors declare that the research was conducted in the absence of any commercial or financial relationships that could be construed as a potential conflict of interest.
