# Supplementary material for: The Sexual Development, Sexual Health, Sexual Experiences, and Sexual Knowledge of Forensic Mental Health Patients: A Research Design and Methodology Protocol
Source: Front Psychiatry. 2021 Jun 4;12:651839. doi: 10.3389/fpsyt.2021.651839 (PMC8212926; doi:10.3389/fpsyt.2021.651839)
Supplement: Supplementary file 1 [file Data_Sheet_1.PDF]

General Sexual Knowledge Questionnaire

Screening Question

1. **Have you ever have any sex education?**

   Mark only one oval.

   - ◯ Yes
   - ◯ No
   - ◯ Can not remember
   - ◯ Other: ______________________

2. **By whom?**

   Check all that apply.

   - ☐ Parents
   - ☐ Peers
   - ☐ School
   - ☐ TV
   - ☐ Other

3. **Have you ever had a discussion about sex related matters with anyone?**

   Mark only one oval.

   - ◯ Yes
   - ◯ No
   - ◯ Maybe

4. **With whom?**

   Check all that apply.

   - ☐ Parents
   - ☐ Peers
   - ☐ School / Educational staff
   - ☐ Medical / health care staff
   - ☐ Others

Physiology: Pictures

I am now going to show you a picture of a man. I would like you to put a cross on the part of the body I describe. If you are not sure about the word I am using please tell me. There are lots of words used to describe parts of the body and you may use a different word to describe that part of the body.

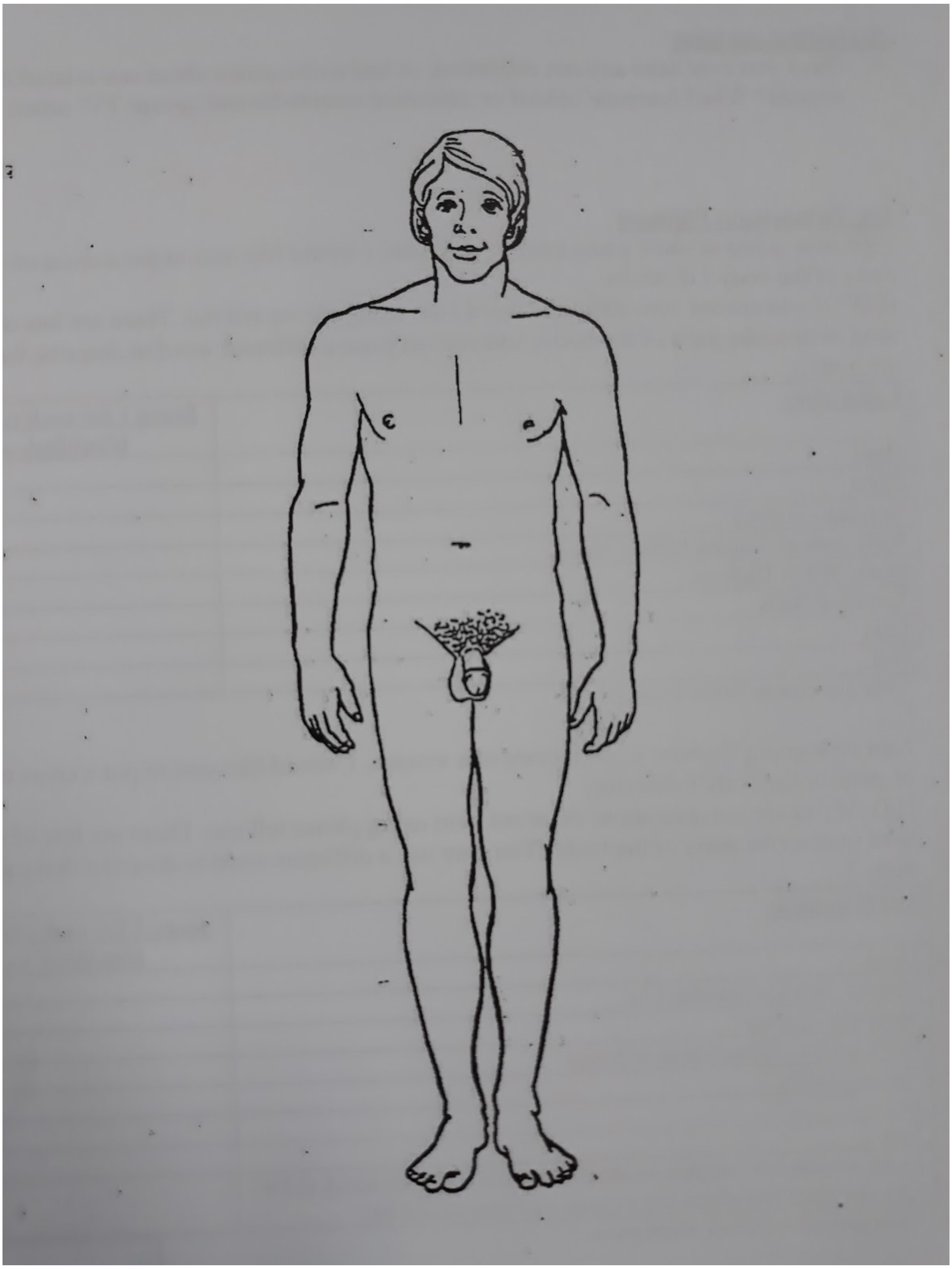

5. **Label man:**

   Check all that apply.

- [ ] Head
- [ ] Chest
- [ ] Stomach / Tummy
- [ ] Belly Button / Tummy Button / Navel
- [ ] Penis / Dick / Willy etc
- [ ] Testicles / Balls
- [ ] Legs
- [ ] Feet

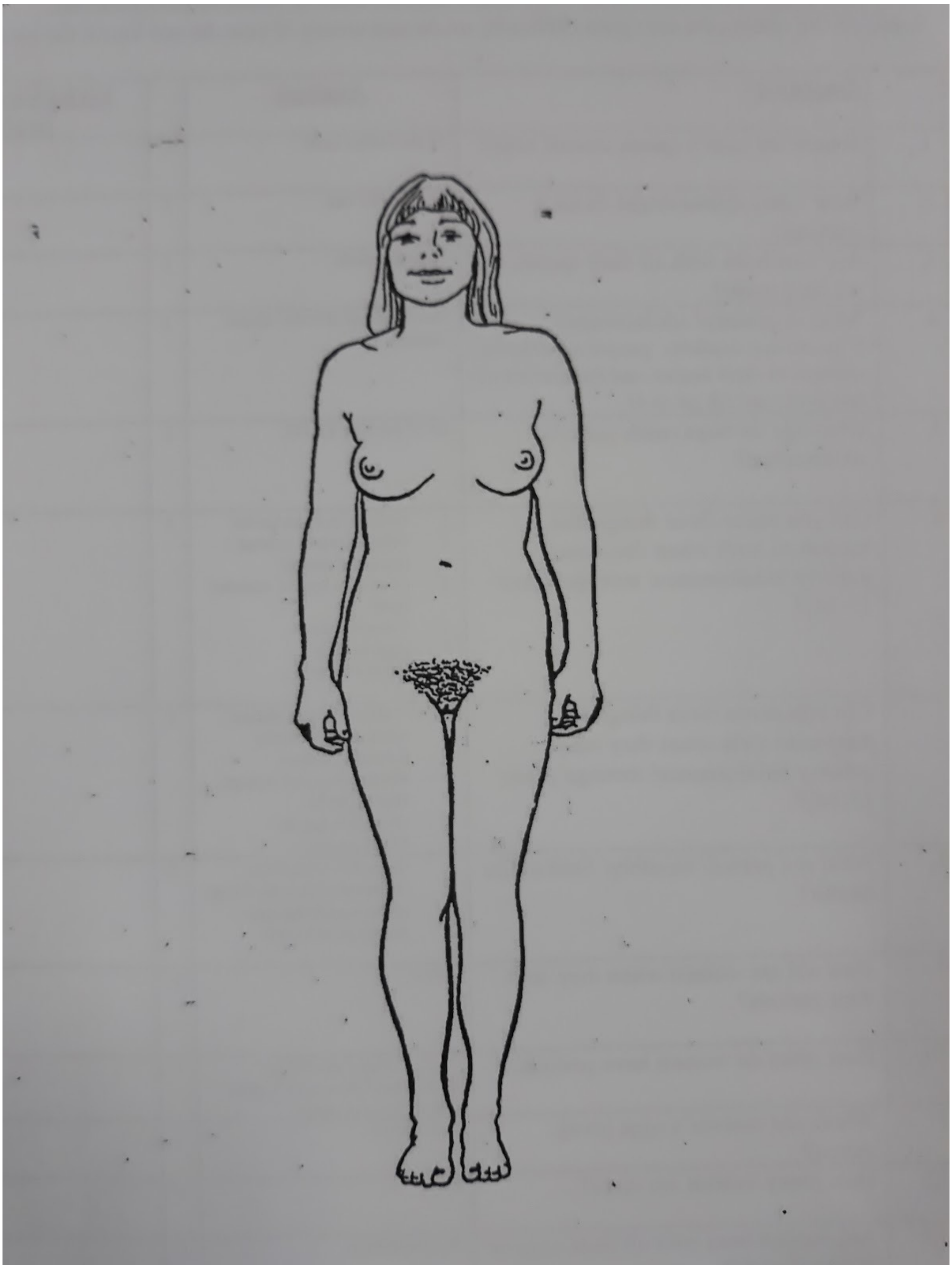

6. **Label for woman:**

   Check all that apply.

   - [ ] Head
   - [ ] Breats / Bosoms / Boobs / Tits
   - [ ] Stomach / Tummy
   - [ ] Belly Button / Tummy Button / Navel
   - [ ] Vagina / Fanny
   - [ ] Legs
   - [ ] Feet
   - [ ] Women have organs inside their bodies called ovaries. Can you show me where the ovaries would be?

Physiology Questions

These are some questions about men and women. answer them the best you can. some of the questions are quite difficult, so dont worry if you do not know the answer.

7. **Where are men's sperm stored?**

   Check all that apply.

   - [ ] Tick box if Answer: In Testes/Balls

8. **How many sperm might fit on a pinhead?**

   Check all that apply.

   - [ ] Tick box if Answer: Thousands / Lots

9. **Are men born with all their sperms or are they made?**

   Check all that apply.

   - [ ] Tick box if Answer: They are made

10. **What is puberty / adolescence? If no answer explain "people experience changes in their bodies and themselves as they grow up and skip next question)**

    Check all that apply.

    - [ ] Tick box if Answer: Development of body / Sexual maturity

11. **What age do boys reach puberty / adolescence?**

    Check all that apply.

    - [ ] Tick box if answer 10-18 (usually 13-14)

12. **Can you name 3 things that happen to boys when they reach puberty? (adolescence, teenage years, 13-14)**
Check all that apply.

- [ ] Increase in testosterone
- [ ] Penis / scrotum enlarge (sexual maturity)
- [ ] Growth in height / muscle / bone
- [ ] Voice breaks
- [ ] Hair on body
- [ ] Mood swings

13. **Can you name 3 things that happen to girls when they reach puberty? (adolescence, teenage years, 13-14)**
Check all that apply.

- [ ] Uterus / Ovaries / Uterine tubes reach maturity
- [ ] Breasts develop / enlarge
- [ ] Hair on body
- [ ] Growth in height
- [ ] Mood swings

14. **What is a period / time of the month?**
Check all that apply.

- [ ] When the ovum is not fertilised
- [ ] When the lining of the womb changes
- [ ] Discharge of blood

15. **How old are women when they start their periods?**
Check all that apply.

- [ ] tix box if answer 10-18 years

16. **How often do women have periods?**
Check all that apply.

- [ ] Tick box if answer once a month / every 26 to 30 days

17. **Where are a women's eggs (ova) stored?**
Check all that apply.

- [ ] Tick box if answer the ovaries

18. **How many ovaries are there?**
Check all that apply.

- [ ] tick the box if answer 2

19. **Are women born with all their eggs or are they made?**
Check all that apply.

- [ ] Tick the box if answer - born with them.

Sexual Intercourse

These are some questions about sexual intercourse or having sex. answer them as best as you can. Some of the questions are quite difficult, so do not worry if you don't know the answer.

20. **SCREEN: Can you tell me what intercourse / having sex is?**
Check all that apply.

- [ ] Tick box if answer is - when the penis is put inside the vagina. also include oral / anal sex
- [ ] Tick box if further explanation were required

21. **What is a virgin?**
Check all that apply.

- [ ] Tick box if answer is - someone who has not had sex / intercourse

22. **Can you have sex when a woman is having a period?**
Mark only one oval.

- ( ) Yes
- ( ) No
- ( ) Maybe

23. **What is an erection (hard on)?**
Check all that apply.

- [ ] Tick box if answer - penis becoming rigid during sexual excitement

24. **What is ejaculation (coming)?**
Check all that apply.

- [ ] Expulsion of semen / when a man comes (during sex / at night / maturbation)

25. **What is impotence?**
Check all that apply.

- [ ] Tick box if answer inability to achieve an erection

26. **What is a prostitute / lady or man on street?**
Check all that apply.

- [ ] Tick box if answer - a person who has intercourse / sex for money / sex industry worker

27. **What is incest?**
Check all that apply.

- [ ] Tick box if answer - intercourse / sex with an immediate relative

Masturbation

28. **SCREEN: What is masturbation / wanking / jerking off / play with self?**
Check all that apply.

- [ ] Tick the box if answer self-stimulation of the genitals
- [ ] Tick the box if further explanation were required

29. **Does masturbation / wanking / jerking off / playing with self have any negative / bad effects for your health?**
Mark only one oval.

- ( ) Yes
- ( ) No
- ( ) Maybe

Pregnancy

These are some questions about pregnancy. Answer them as best as you can. Some of the questions are quite difficult, so don't worry if you do not know the answer.

30. **Screen: Can you tell me what pregnant means?**
Check all that apply.

- [ ] Tick the box if answer: When a woman is going to have a baby.
- [ ] Tick the box if further explanation were required

31. **How do a women become pregnant?**
Check all that apply.

- [ ] By having sexual intercourse
- [ ] Sperm fertilise eggs (including IVF)

32. **How long does it take from getting pregnant to having a baby?**
Check all that apply.

- [ ] Tick box if answer 9 months

33. **How does a women know that she is pregnant?**
Check all that apply.

- [ ] No periods / period stops
- [ ] Pregnancy test / GP
- [ ] Morning sickness / nausea
- [ ] Heavy, tender breasts
- [ ] Vaginal discharge / irritation
- [ ] Cravings
- [ ] Stomach is bigger
- [ ] Hormone changes - mood swings, temperature changes
- [ ] Tiredness
- [ ] Sense of knowing

34. **When is the risk of pregnancy least during a woman's monthly cycle?**
Check all that apply.

- [ ] Tick box if answer: when they are having a period (approx 2%)

35. **From what part of a woman does the baby come out?**
Check all that apply.

- [ ] Tick box if answer - Vagina / womb / uterus

36. **What is the belly button / tummy button / navel?**
Check all that apply.

- [ ] Tick box if answer: remains of the cord connecting mother and baby.

37. **When is the risk of pregnancy highest during a woman's monthly cycle?**
Check all that apply.

- [ ] Tick box if answer on day 7 - 14 of her cycle

Contraception

These are some questions about contraception. Answer them as best as you can. Some of the questions are quite difficult, so don't worry if you do not know the answer.

38. **SCREEN: Can you tell me what is birth control / contraception?**
Check all that apply.

- [ ] Tick the box if answer: method of stopping fertilisation / having babies
- [ ] Tick the box if further explanation were required

39. **Can you name three types of contraception:**
Check all that apply.

- [ ] Abstinence / not having sex
- [ ] Cap / diaphragm
- [ ] Coil / IUD/ Loop
- [ ] Condom
- [ ] Contrceptive pill
- [ ] Foam / Jelly / Cream
- [ ] Injection / Depo Provera
- [ ] Implant
- [ ] Natural / Rythm / Safe days
- [ ] Oral / anal sex
- [ ] Sterilisation for female
- [ ] Vasectomy for male
- [ ] Withdrawal

40. **Can you explain how they work?**
Mark only one oval per row.

	Yes	No	Stops babies
Method 1	◯	◯	◯
Method 2	◯	◯	◯
Method 3	◯	◯	◯

41. **Can you name three places where you might buy / be given contraceptives?**
Check all that apply.

- [ ] Family planning clinic
- [ ] Health Centre
- [ ] Other clinic
- [ ] GP
- [ ] Hospital
- [ ] Private Hospital / Clinic / GP
- [ ] Chemist / Pharmacy
- [ ] Other shop / garage / pubption 7
- [ ] Community Health worker
- [ ] Friend / Relative / Care worker

42. **Can you name any other forms of contraception:**
Check all that apply.

- [ ] Abstinence / not having sex
- [ ] Cap / diaphragm
- [ ] Coil / IUD/ Loop
- [ ] Condom
- [ ] Contrceptive pill
- [ ] Foam / Jelly / Cream
- [ ] Injection / Depo Provera
- [ ] Implant
- [ ] Natural / Rythm / Safe days
- [ ] Oral / anal sex
- [ ] Sterilisation for female
- [ ] Vasectomy for male
- [ ] Withdrawal

Sexually Transmitted Diseases

These are some questions about STD's / Sexually transmitted infections / Venereal diseases. Answer them as best as you can. Some of the questions are quite difficult, so don't worry if you do not know the answer.

43. **SCREEN: What is an STD/STI/Venereal disease?**
Check all that apply.

- [ ] Tick box if further explanation required.
- [ ] Tick box if answer: Infection or disease that has been transmitted through intercourse / sex
- [ ] Tick box if answer: Infection or disease that has been transmitted by having unprotected sex (penetrative / anal / oral)

44. **Can you name five STD's?**
Check all that apply.

- [ ] Chlamydia
- [ ] Herpes Simplex
- [ ] Gonorrhoea / Clap
- [ ] Trichomoniasis
- [ ] Syphilis / Pox
- [ ] Genitall warts
- [ ] HIV / AIDS
- [ ] Hepatitis B or C
- [ ] Thrush
- [ ] Crab / Lice

45. **Can you name any symptoms you might get if you had an STD?**
Check all that apply.

- [ ] Damage to the immune system
- [ ] Soreness
- [ ] Itching
- [ ] Blisters / Sores / Warts
- [ ] Pain when passing urine
- [ ] Pain when having sex
- [ ] Discharge (smell)
- [ ] Flu-like symtpoms
- [ ] Death
- [ ] Don't always have symtpoms

46. **Which contraceptives can help prevent STD's?**
Check all that apply.

- [ ] Tixck box if answer: condom

47. **What is HIV?**
Check all that apply.

- [ ] Human immunodeficiency virus
- [ ] Vius that attacks the immune system

48. **What is the difference between HIV / AIDS?**
Check all that apply.

- [ ] Tick box if answer: a person has AIDS when HIV weakens the body so much that certain illness develop

49. **How might someone become infected with HIV?**
Check all that apply.

- [ ] Sexual intercourse / vaginal / anal / oral
- [ ] Exposure to blood - injection, transfusion or non sterile equipment
- [ ] mother to baby - womb, breastmilk

50. **How can you protect yourself from the risk of getting HIV?**
Check all that apply.

- [ ] Sex using a condom
- [ ] Non penetrative sex
- [ ] Sterile needles

Sexuality

These are some questions about sexuality. Answer them as best as you can. Some of the questions are quite difficult, so don't worry if you do not know the answer.

51. **SCREEN: What is a homosexual (gay/lesbian)**
Check all that apply.

- [ ] tick box if answer: someone who is attracted to people of the same sex.
- [ ] tick if further explanation were required

52. **What is a heterosexual (straight)?**
Check all that apply.

- [ ] Tick box if answer: someone who is attracted to people of the opposite sex.

53. **What is a bisexual ?**
Check all that apply.

- [ ] Tick box if answer: someone who is attracted to people of the same or opposite sex.

Wrap up

That is the end of the questions. Thank you.
Do you have any questions or would you like for any information on any of the topics we have talked about today?
