# Supplementary material for: The Sexual Development, Sexual Health, Sexual Experiences, and Sexual Knowledge of Forensic Mental Health Patients: A Research Design and Methodology Protocol
Source: Front Psychiatry. 2021 Jun 4;12:651839. doi: 10.3389/fpsyt.2021.651839 (PMC8212926; doi:10.3389/fpsyt.2021.651839)
Supplement: Supplementary file 2 [file Data_Sheet_2.PDF]

Participant Questionnaire - Let's talk about sex

Table of Contents

Section 1- Demographics....1

Section 2 - Sexual development....3

Section 3 - Physical Development....6

Section 4 - Emotional experiences....7

Section 5 - Sexual identity....8

Section 6 - First sexual experiences....10

Section 7 - Female sexual partners - all....12

Section 8 - Regular female sexual partner....13

Section 9 - Most recent female partner....15

Section 10 - Sex with Men - all partners....18

Section 11 - Regular male partners....19

Section 12 - Most recent male partner....20

Section 13 - Masturbation, alternative and other non coital sexual behaviour....24

Section 14 - Sex and the internet....26

Section 15 - Sex Work....28

Section 16 - Sexual coercion....29

Section 17 - Domestic violence....30

Section 18 - Sexual difficulties:....31

Section 19 - Contraception....36

Section 20 - Sexual transmissible disease / infection....39

Section 21 - Body Modification....41

Section 22 - Sex education....42

Section 23 - Sexual knowledge....43

Section 24 - Sexual attitudes....44

Section 25 - General health 45

Section 26 - Alcohol and Substances 47

Section 27 - Vocational / Educational questions 49

Section 28 - Mental health - attitudes 51

Section 29 - Quality of life 52

Section 30 - Sexual Dysfunction Questionnaire 53

Section 31 - Anti-psychotic and Sexual Functioning Questionnaire (ASFQ) 54

Section 32 - Brief Psychiatric Rating Scale 58

Section 33 MARS – Medication Adherence Rating Scale: 59

Section 34 - Wrap up 60

Participant Questionnaire - Let's talk about sex.

Section 1- Demographics

*Required

1. **Participant identification number? ***

2. **Date of Birth? ***

Example: December 15, 2012

3. **Country of Birth?**

4. **Language spoken at home?**

5. **Postcode for place of residence?**

6. **Legal marital status?**

Mark only one oval.

- ◯ Never married
- ◯ Widowed
- ◯ Divorced
- ◯ Separated not divorced
- ◯ Married
- ◯ Civil / Registered partnership
- ◯ Other: ______

7. **Co-residing in Household?**

Check all that apply.

- [ ] Spouse / Partner
- [ ] Children <5 years of age
- [ ] Children 5-15 years
- [ ] Other family?¶
- [ ] Other people?¶

8. **How many children have you had?**

____

9. **Do you have a particular religion or faith?**

Mark only one oval.

- ( ) Yes
- ( ) No
- ( ) Maybe

Section 2 - Sexual development

10. **Have you ever been kissed?**

Mark only one oval.

- Yes
- No

11. **Age at first kiss?**

12. **Total number of kissing partners?**

13. **How long ago was the last time you have been kissed?**

14. **Have you ever touched your partner's genitals with your hands?**

Mark only one oval.

- Yes
- No

15. **Age at first touching your partners genitals with your hands?**

16. **Total number of partners that's genitals you touched?**

17. **How long ago was the last time you touched a partners genitals?**

18. **Have you ever been touched on your genitals by a partner?**

Mark only one oval.

- Yes
- No

19. **Age at first being touched by a partner on your genitals with their hands?**

20. **Total number of partners that touched your genitals**

21. **How long ago was the last time you have been touched on your genitals by a partner**

22. **Have you ever given someone oral sex?**

Mark only one oval.

- Yes
- No

23. **Age at first giving oral sex experience?**

24. **Total number of partners you gave oral sex to?**

25. **How long ago was the last time you gave someone oral sex?**

26. **Have you ever received oral sex?**

Mark only one oval.

- Yes
- No

27. **Age at first receiving oral sex experience?**

28. **Total number of partners that you received oral sex from**

29. **How long ago was the last time you received oral sex?**

30. **Have you ever had intercourse without a condom?**

Mark only one oval.

- ( ) Yes
- ( ) No

31. **Age at first intercourse without a condom experience?**

____

32. **Total number of partners you had intercourse with, without a condom**

____

33. **How long ago was the last time you had intercourse without a condom?**

____

34. **Have you ever had intercourse with a condom?**

Mark only one oval.

- ( ) Yes
- ( ) No

35. **Age at first intercourse with a condom experience?**

____

36. **Total number of partners you had intercourse with, with a condom**

____

37. **How long ago was the last time you had intercourse with a partner with a condom?**

____

Section 3 - Physical Development

38. **Do you believe your physical development has been normal?**

Mark only one oval.

- Yes
- No

39. **Have you ever seen a doctor due to late development?**

Mark only one oval.

- Yes
- No

40. **Age at first ejaculation?**

*

____

*

Section 4 - Emotional experiences

41. **After the last time you had sex, to what extent did you feel?**

Mark only one oval per row.

	1 Not at all,	2.	3.	4.	5. Extremely
Good	◯	◯	◯	◯	◯
Upset	◯	◯	◯	◯	◯
Guilty	◯	◯	◯	◯	◯
Happy	◯	◯	◯	◯	◯
Used	◯	◯	◯	◯	◯
Fantastic	◯	◯	◯	◯	◯
Worried	◯	◯	◯	◯	◯
Loved	◯	◯	◯	◯	◯
Regretful	◯	◯	◯	◯	◯
Abused¶	◯	◯	◯	◯	◯

Section 5 - Sexual identity

42. **What do you consider your gender identity to be?**

Mark only one oval.

- ◯ Male
- ◯ Female
- ◯ Gender fluid
- ◯ No binary
- ◯ Intersex
- ◯ Other
- ◯ Not sure

43. **What do you consider your sexual identity to be?**

Mark only one oval.

- ◯ Heterosexual / Straight
- ◯ Homosexual / Gay / Lesbian
- ◯ Bisexual
- ◯ Not sure / Undecided
- ◯ Something else

44. **Which of these 6 statements describe you best?**

Mark only one oval.

- ◯ I felt sexually attracted only to females, never to males
- ◯ ……. More often to females, and at least once to a male
- ◯ …….About equally often to females and males
- ◯ ……. More often to males, and at least once to a female
- ◯ ……. Only to males, never to females
- ◯ I have never felt sexually attracted to anyone at all

45. **In the next question, sexual experience means any kind of contact with another person that you felt was sexual. This could be kissing, touching, intercourse or any other form of sex. Which of these 6 statements describe you best?**

- ◯ I have had sexual experiences with females, never to males
- ◯ .... More often with females, and at least once with a male
- ◯ ....About equally often with females and males
- ◯ .... More often with males, and at least once with a female
- ◯ .... Only with males, never with females
- ◯ I have never had any sexual experience with anyone at all

Section 6 - First sexual experiences

46. **How old were you when you first had vaginal intercourse (not Child Sexual Abuse)**

____

47. **How old was she?**

____

48. How long had you know her before you had sex for the first time?

Mark only one oval.

- ( ) Less than 24hrs
- ( ) 1-7days
- ( ) 7days to 1 month
- ( ) 1 month to 1 year
- ( ) More than 1 year

49. **What was your relationship to her?**

Mark only one oval.

- ( ) Wife
- ( ) Fiancée
- ( ) Living together
- ( ) Steady partner / girlfriend
- ( ) Casual partner
- ( ) Sex worker

50. **What contraception / precaution did you use?**

____

51. **How old were you when you first had oral sex with a female?**

____

52. **Have you ever had anal sex with a woman?**

Mark only one oval.

- ( ) Yes
- ( ) No

53. **How old were you when you first had sex with a male?**

54. **How old was he?**

55. **How long had you know him before you had sex for the first time?**

Mark only one oval.

- Less than 24hrs 1-
- 7days
- 7days to 1 month
- 1 month to 1 year
- More than 1 year

56. **What was your relationship to him?**

- Living together
- Steady partner / boyfriend
- Casual partner
- Sex worker

Section 7 - Female sexual partners - all

57. **In your whole life how many women have you had vaginal or anal intercourse with?**

58. **In the last 5 years how many women have you had vaginal or anal intercourse with?**

59. **In the last 12 months how many women have you had vaginal or anal intercourse with?**

60. **How many women did you have oral sex with?**

61. **In the last 12 months, how many women did you have oral sex with?**

62. **How many women have you had some form of sexual contact with that involved stimulating the penis or vaginal area?**

63. **How many women that you had some form of sexual contact with that involved stimulating the penis or vaginal area in the last 12 months?**

Section 8 - Regular female sexual partner

Questions about regular partner you most recently had sex with.

64. **Do you currently have a regular female sexual partner / partners?**

Mark only one oval.

- Yes
- No

65. **How many regular female sexual partner / partners do you have?**

____

66. **Do you live with any of your regular sexual partner?**

Mark only one oval.

- Yes
- No

67. **Questions about regular partner you most recently had sex with. How long have you been in this relationship?**

____

68. **How long did you know her before you had sex?**

____

69. **How old was she?**

____

70. **In this relationship, do you expect that your partner would have sex only with you?**

Mark only one oval.

- Yes
- No
- Maybe

71. **In this relationship, do you expect that you would have sex only with her?**

- Yes
- No
- Maybe

72. **Have you discussed these expectations with her?**

Mark only one oval.

- Yes
- No
- Maybe

73. **Have you both explicitly agreed about this?**

Mark only one oval.

- Yes
- No
- Maybe

74. **How many times is past 4 weeks have you had sex with your partner?**

____

75. **How physically pleasurable do you find sex with her to be?**

Mark only one oval.

- Extremely pleasurable
- Very pleasurable
- Moderately pleasurable
- Slightly pleasurable
- Not at all pleasurable

76. **How emotionally satisfying do you find your relationship with her?**

Mark only one oval.

- Extremely
- Very
- Moderately
- Slightly Not
- at all

Section 9 - Most recent female partner

77. **When was the last time you had sex with this woman?**

78. **What was your relationship with her?**

Mark only one oval.

- Live in partner
- Regular partner – not living in
- Occasional partner
- Casual partner
- Other

79. **How old was she?**

80. **How long did you know her before you had sex?**

81. **How long ago was the first time you had sex with her?**

82. **How many times did you have sex with her in the past 4 weeks?**

83. **Did you have vaginal intercourse the last time you had sex with her?**

Mark only one oval.

- Yes
- No

84. **If yes, Did you ejaculate inside her vagina?**

Mark only one oval.

- Yes
- No

85. **Did you have anal intercourse with her?**

Mark only one oval.

- ⬭ Yes
- ⬭ No

86. **If yes, Did you ejaculate inside her rectum?**

Mark only one oval.

- ⬭ Yes
- ⬭ No

87. **Did she have oral sex with you (your penis in her mouth)?**

Mark only one oval.

- ⬭ Yes
- ⬭ No

88. **Did you have oral sex with her (your mouth on her vaginal area)?**

Mark only one oval.

- ⬭ Yes
- ⬭ No

89. **Did she stimulate your penis with her hand?**

Mark only one oval.

- ⬭ Yes
- ⬭ No

90. **Did you stimulate her clitoris / vaginal area with your hand?**

Mark only one oval.

- ⬭ Yes
- ⬭ No

91. **The last time you had sex, did you have an orgasm?**

Mark only one oval.

- Yes
- No

Section 10 - Sex with Men - all partners

92. **How many men have you had anal intercourse with?**

93. **In the last 5 years, how many men have you had anal intercourse with?**

94. **In the last 12 months, how many men have you had anal intercourse with?**

95. **How many men have you had oral sex with (his penis in your mouth / your penis in his mouth)?**

96. **In the last 5 years, how many men have you had oral sex with?**

97. **In the last 12 months, how many men have you had oral sex with?**

98. **How many men have you had any other form of sexual contact with (touching the penis)?**

99. **In the last 12 months, how many men have you had any other form of sexual contact with (touching the penis)?**

Section 11 - Regular male partners

100. **Do you have a regular male sexual partner?**

Mark only one oval.

- Yes
- No

101. **How many regular male partners do you have?**

____

102. **Do you live with him?**

Mark only one oval.

- Yes
- No

103. **How long have you been in this relationship?**

____

104. **How long had you known him before you had sex for the first time?**

____

105. **How old is he?**

____

106. **In this relationship, do you expect that your partner would have sex only with you?**

Mark only one oval.

- Yes
- No
- Maybe

107. **In this relationship, do you expect that you would have sex only with him?**

Mark only one oval.

- Yes
- No
- Maybe

108. **Have you discussed these expectations with him?**

Mark only one oval.

- Yes
- No
- Maybe

109. **Have you both explicitly agreed about this?**

Mark only one oval.

- Yes
- No
- Maybe

110. **How many times is past 4 weeks have you had sex with your male partner?**

____

111. **How physically pleasurable do you find sex with him to be?**

Mark only one oval.

- Extremely pleasurable
- Very pleasurable
- Moderately pleasurable
- Slightly pleasurable
- Not at all pleasurable

112. **How emotionally satisfying do you find your relationship with him?**

Mark only one oval.

- Extremely
- Very
- Moderately
- Slightly Not
- at all

Section 12 - Most recent male partner

113. **When was the last time you had sex with this man?**

____

114. **What was your relationship with him?**

Mark only one oval.

- ( ) Live in partner
- ( ) Regular partner – not living in
- ( ) Occasional partner
- ( ) Casual partner
- ( ) Other

115. **How old was he?**

____

116. **How long did you know him before you had sex?**

____

117. **How long ago was the first time you had sex with him?**

____

118. **How many times did you have sex with him in the past 4 weeks?**

____

119. **The last time you had sex; did you put your penis into his anus?**

Mark only one oval.

- ( ) Yes
- ( ) No

120. **Did you ejaculate inside his rectum?**

Mark only one oval.

- ( ) Yes
- ( ) No

121. **The last time you had sex; did he put his penis into your anus?**

Mark only one oval.

- Yes
- No

122. **Did he ejaculate inside your rectum**

Mark only one oval.

- Yes
- No

123. **Last time you had sex; did you have oral sex – his mouth on your penis?**

Mark only one oval.

- Yes
- No

124. **Last time you had sex; did you have oral sex – your mouth on your penis?**

Mark only one oval.

- Yes
- No

125. **Did he stimulate your penis with his hand?**

Mark only one oval.

- Yes
- No

126. **Did you stimulate his penis with your hand?**

Mark only one oval.

- Yes
- No

127. **The last time you had sex, did you have an orgasm?**

Mark only one oval.

- Yes
- No

Section 13 - Masturbation, alternative and other non coital sexual behaviour

128. **Have you ever looked at pornographic material?**

Mark only one oval.

- Yes
- No

129. **Have you looked at pornographic material in the past 12 months?**

Mark only one oval.

- Yes
- No

130. **How old were you when you first looked at pornographic material?**

____

131. **Answer the following**

Mark only one oval per row.

	Strongly Agree		Neither agree nor Disagree		Strongly disagree
Pornography can enhance the pleasure of masturbation					
You feel using pornography has had a bad effect on you					
You feel that you are addicted to porn					

132. **In the past 12 months have you....**

Mark only one oval per row.

	Yes	No
Have you ever masturbated alone?	◯	◯
Watched an X-rated movie?	◯	◯
Used a sex toys during sex with a partner?	◯	◯
Been involved in role play or dressing up?	◯	◯
Have you been involved with BDSM?	◯	◯
Have you been involved in group sex?	◯	◯
Have you been involved in swinging or partner swapping?	◯	◯
Have you used your fingers to stimulate a partner's anus, or had a partner do that to you?	◯	◯
Have you been involved in fisting?	◯	◯
Have you been involved in rimming – Oral-anal contact?	◯	◯

Section 14 - Sex and the internet

133. **Have you ever used an internet site or a smart phone application to look for potential partners?**

Mark only one oval.

- Yes
- No

134. **Have you done so in the past year?**

Mark only one oval.

- Yes
- No

135. **Have you had online sex with someone in the past year?**

Mark only one oval.

- Yes
- No

136. **In the last year, have you met someone in person that you met first on an internet site?**

Mark only one oval.

- Yes
- No

137. **How many?**

____

138. **Did you have sex with anyone you have first met on an internet site?**

Mark only one oval.

- Yes
- No

139. **The most recent time was that person male or female?**

Mark only one oval.

- ◯ Female
- ◯ Male

Section 15 - Sex Work

140. **Answer the following questions with yes / no**

Mark only one oval per row.

	Yes	No
Have you ever paid anyone to have sex with you (including oral / manual stimulation)?		
2. Have you ever paid for sex with a woman?		
3. Have you ever paid for sex with a woman in the past 12 months?		
4. Have you ever paid for sex with a woman, overseas, in the past 12 months?		
5. Have you ever paid for sex with a man?		
6. Have you ever paid for sex with a man in the past 12 months?		
7. Have you ever paid for sex with a man, overseas, in the past 12 months?		
8. Have you ever been paid to have sex (including oral / manual stimulation)?		
9. Has a man paid money for sex with you in the last 12 months?		
10. Has a women paid for sex with you in the last 12 months?		

Section 16 - Sexual coercion

141. **Have you ever had a sexual experience with a male/female partner when you didn't want to because you were to drunk or high at the time?**

Mark only one oval.

- Yes
- No
- Maybe

142. **Have you ever been forced or frightened by a male / female into doing something sexually that you did not want to do?**

Mark only one oval.

- Yes
- No
- Maybe

143. **How many times did it happen?**

____

144. **How old were you the first time when it started?**

____

145. **Did you talk to someone or seek help?**

Mark only one oval.

- Yes
- No

Section 17 - Domestic violence

146. **During the past 12 months, how often has your regular partner physically hurt you?**

147. **During the past 12 months, how often has your regular partner insulted or talked down to you?**

148. **During the past 12 months, how often has your regular partner threatened you with harm?**

149. **During the past 12 months, how often has your regular partner screamed or sworn at you?**

Section 18 - Sexual difficulties:

150. **During the last year has there been a period of one month or more when you lacked interest in having sex?**

Mark only one oval.

- Yes
- No
- Maybe

151. **How long did it lasts?**

- Less than one month 1-
- 3months
- 3-6months
- >6 months

152. **How much of a problem was this?**

Mark only one oval.

- Not a problem
- Minor problem
- Somewhat
- Major problem

153. **Did you seek treatment for it?**

Mark only one oval.

- Yes
- No

154. **Has there been a period of one month or more when you were unable to come / orgasm?**

Mark only one oval.

- Yes
- No
- Maybe

155. **How long did it lasts?**

Mark only one oval.

- Less than one month 1-
- 3months
- 3-6months
- >6 months

156. **How much of a problem was this?**

Mark only one oval.

- Not a problem
- Minor problem
- Somewhat
- Major problem

157. **Did you seek treatment for it?**

Mark only one oval.

- Yes
- No

158. **Has there been a period of one month or more when you come to orgasm to quickly?**

Mark only one oval.

- Yes
- No
- Maybe

159. **How long did it lasts?**

Mark only one oval.

- Less than one month 1-
- 3months
- 3-6months
- >6 months

160. **How much of a problem was this?**

Mark only one oval.

- Not a problem
- Minor problem
- Somewhat
- Major problem

161. **Did you seek treatment for it?**

Mark only one oval.

- Yes
- No

162. **Has there been a period of one month or more when you felt anxious about your ability to perform sexually?**

Mark only one oval.

- Yes
- No
- Maybe

163. **How long did it lasts?**

Mark only one oval.

- Less than one month 1-
- 3months
- 3-6months
- >6 months

164. **How much of a problem was this?**

Mark only one oval.

- Not a problem
- Minor problem
- Somewhat
- Major problem

165. **Did you seek treatment for it?**

Mark only one oval.

- Yes
- No

166. **Has there been a period of one month or more when you had trouble keeping an erection when you wanted to?**

Mark only one oval.

- Yes
- No
- Maybe

167. **How long did it lasts?**

Mark only one oval.

- Less than one month 1-
- 3months
- 3-6months
- >6 months

168. **How much of a problem was this?**

Mark only one oval.

- Not a problem
- Minor problem
- Somewhat
- Major problem

169. **Did you seek treatment for it?**

Mark only one oval.

- Yes
- No

170. **Have you ever used any treatment to help with erections?**

Mark only one oval.

- ◯ Yes
- ◯ No

171. **What treatment?**

____

172. **Have you used any treatments in the past 12 months?**

Mark only one oval.

- ◯ Yes
- ◯ No

173. **How was this treatment obtained?**

Mark only one oval.

- ◯ With a doctor's prescription?
- ◯ Online, without a prescription?
- ◯ Some other way?

174. **Ideally, how often would you like to have sex?**

____

Section 19 - Contraception

175. **Before you had sex the last time, did you talk to this person about**

Mark only one oval per row.

	Yes	No
a. Avoiding pregnancy		
b. Avoiding HIV		
c. Avoiding STD		
d. How to get sexual pleasure without intercourse		
e. Using a condom		

176. **Did you use contraception with your regular (most recent) partner?**

Mark only one oval.

- Yes
- No

177. **What form of contraception?**

Check all that apply.

- a. Contraceptive pill
- b. IUD
- c. Depo injection
- d. Implant
- e. Vasectomy
- f. Tubal ligation
- g. Condom
- h. Safe period / Rhythm method
- i. Withdrawal / Coitus interruptus
- j. Nuva-ring
- k. Diaphragm / Cervical cap
- l. Spermicide foam or jelly
- m. Breastfeeding
- n. Other

178. **Have you been having sex without falling pregnant for the past 12 months?**

Mark only one oval.

- Yes
- No
- Maybe

179. **If no contraception, why not?**

____

180. **Condom use during last female sexual experience?**

Mark only one oval per row.

	Yes	No
a. Do you use a condom during vaginal intercourse?		
b. Did you put the condom on before your penis touched her vagina?		
c. Do you use a condom during anal intercourse?		
d. Did you put the condom on before your penis touched her anus?		
e. Do you use a condom during oral intercourse?		

181. **How often was a condom used in past 6 months when having vaginal intercourse with your regular female partner (s)?**

- Never
- Occasionally
- Often
- Always

182. **How often was a condom used in past 6 months when having vaginal intercourse with any other female partner (s)?**

Mark only one oval.

- Never
- Occasionally
- Often
- Always

183. **Have you used condoms in the past to have sex with a male?**

Mark only one oval per row.

	Yes	No
Never		
In the past 12 months		

184. **How often was a condom used when having anal intercourse with your regular male sexual partner?**

- Never
- Occasionally
- Often
- Always

185. **Was the condom put on before his penis touched your anus?**

Mark only one oval.

- Yes
- No

186. **Is your partner HIV +?**

- Yes
- No
- Don't know

Section 20 - Sexual transmissible disease / infection

187. **Have you been tested for a STD in the past year?**

Mark only one oval.

- Yes
- No

188. **How many times?**

____

189. **The last time did it involve**

Check all that apply.

- [ ] a. Urine test
- [ ] b. Blood test
- [ ] c. Throat swab
- [ ] d. Anal swab
- [ ] e. Some other test

190. **STD infection - have you ever had**

Check all that apply.

- [ ] Pubic lice / crabs
- [ ] Genital warts
- [ ] Anal warts
- [ ] Chlamydia
- [ ] Genital Herpes
- [ ] Syphilis
- [ ] Gonorrhoea
- [ ] Non-specific urethritis
- [ ] Candida / thrush
- [ ] HIV

191. **In the last 12 months have you had**

Check all that apply.

- ☐ Pubic lice / crabs
- ☐ Genital warts
- ☐ Anal warts
- ☐ Chlamydia
- ☐ Genital Herpes
- ☐ Syphilis
- ☐ Gonorrhoea
- ☐ Non-specific urethritis
- ☐ Candida / thrush
- ☐ HIV

192. **Where did you go for treatment?**

Check all that apply.

- ☐ 1. Usual GP
- ☐ 2. New GP
- ☐ 3. 24-hr clinic
- ☐ 4. Sexual health clinic
- ☐ 5. Public hospital / outpatients
- ☐ 6. Private hospital
- ☐ 7. Family planning clinic
- ☐ 8. Alternative health professional
- ☐ 9. Chemist
- ☐ 10. Friend
- ☐ 11. Self-treated

Section 21 - Body Modification

193. **Have you been circumcised?**

Mark only one oval.

- Yes
- No

194. **How old were you?**

____

195. **In the past 12 months have you had your pubic hair shaved, waxed, lasered?**

Mark only one oval.

- Yes
- No

196. **Have you ever had surgery to extend the length of your penis?**

Mark only one oval.

- Yes
- No

Section 22 - Sex education

197. **Did you receive any sexual education?**

Mark only one oval.

- Yes
- No
- Maybe

198. **From whom?**

____

199. **Did you receive any sex education at school?**

Mark only one oval.

- Yes
- No
- Maybe

200. **Did it include contraception and condom use?**

Mark only one oval.

- Yes
- No
- Maybe

Section 23 - Sexual knowledge

201. **Answer the following true / false questions**

Mark only one oval per row.

	True	False
a. Chlamydia affects only women (False)		
b. Chlamydia can lead to infertility in women (True)		
c. Once a person has genital herpes, they will always have the virus(True)		
d. People who have injected drugs are at risk for hepatitis C (True)		
f. Gonorrhoea can be transmitted through oral sex (True)		
g. Genital warts can only be spread by intercourse (False)		
h. Cold sores and genital Herpes can be caused by the same virus (True)		

Section 24 - Sexual attitudes

202. **Answer the following**

Mark only one oval per row.

	Strongly Agree		Neither agree or Disagree	Disagree	Strongly disagree
a. Sex before marriage is acceptable					
b. If two people had oral sex, but not intercourse, you would still consider that they had sex together					
c. An active sex life is important for your sense of well being					
d. Abortion is always wrong					
e. Having an affair when in a committed relationship is always wrong					
f. Sex between two adult women is always wrong					
g. Sex between two adult men is always wrong					
h. Pornography degrades the women shown in it					
i. Pornography can improve sexual relations amongst adults					
j. Pornography degrades men shown in it					
k. Sexual health education for adolescents increase the likelihood of early sexual activity					
l. People who are suspected of having HIV / AIDS lose respect in the community					
m. It is acceptable for women to continue to work outside the home after they have children, if they want to					

203. **What would upset or distress you more…imagining your partner**

Mark only one oval.

- a. Forming a deep emotional attachment to another person?
- a. Having sexual intercourse with another person?

Section 25 - General health

204. **In general would you say your health is:**

Mark only one oval.

- ◯ Excellent
- ◯ Very good
- ◯ Good
- ◯ Fair
- ◯ Poor

205. **Do you have high blood pressure or a heart condition?**

Mark only one oval.

- ◯ Yes
- ◯ No
- ◯ Maybe

206. **Do you have high blood sugar or diabetes?**

Mark only one oval.

- ◯ Yes
- ◯ No
- ◯ Maybe

207. **Are you taking any medical medication?**

Mark only one oval.

- ◯ Yes
- ◯ No

208. **Have you ever been diagnosed with prostate cancer?**

- ◯ Yes
- ◯ No
- ◯ Maybe

209. **Did your treatment for prostate cancer involved surgery?**

Mark only one oval.

- ( ) Yes
- ( ) No

Section 26 - Alcohol and Substances

210. **Do you smoke cigarettes, cigars, pipes or any other tobacco products?**

Mark only one oval.

- Yes
- No

211. **Would that be**

Mark only one oval.

- a. Daily
- b. At least weekly
- c. Less often than weekly

212. **Over your lifetime would you have smoked more than 100 cigarettes?**

Mark only one oval.

- Yes
- No
- Maybe

213. **For how many years did you smoke?**

214. **How many cigarettes would you have smoked on a day?**

215. **How often do you have an alcoholic drink of any kind?**

216. **When you drink alcoholic drinks, how many would you usually have?**

217. **Answer the following:**

Mark only only one oval per row.

	Yes	No
Have you ever injected any drugs?		
In the past 12 months, have you ever injected any drugs?		
Have you smoked, inhaled any other recreational drugs?		
In the past 12 months did you smoked, inhaled any other recreational drugs?		

218. **Typical drug use when you have sex**

Mark only one oval per row.

	Always	Most of the time	Rarely	Never
Alcohol				
Cannabis				
Amphetamines - Ice Speed				
Opiates – methadone / heroin				
Cocaine				
Hallucinogens LSD				
Other				

Section 27 - Vocational / Educational questions

219. **What is the highest education qualification you have completed?**

Mark only one oval.

- a. No formal schooling
- b. Primary school
- c. Lower secondary school / School certificate / Intermediate certificate
- d. Technical or trade certificate
- e. Higher secondary school / HSC/VCE/Leaving certificate
- f. College certificate / Diploma
- g. Undergraduate university degree
- h. Postgraduate university degree

220. **Which of the following best describe your work status now?**

Mark only one oval.

- a. Employed – full time
- b. Employed – part-time
- c. Home duties
- d. Unemployed
- e. Student
- f. Permanently ill or unable to work
- g. Retired

221. **Have you ever worked?**

Mark only one oval.

- Yes
- No
- Maybe

222. **What is your usual job?**

Mark only one oval.

- a. Manager / Administrator
- b. Professional
- c. Associate professional
- d. Tradesperson or related
- e. Advance clerical and services
- f. Intermediate clerical, sales and services
- g. Intermediate production and transport
- h. Elementary clerical, sales and services
- i. Labourer and related

223. **What is you approximate family income before tax and other deductions?**

Mark only one oval.

- a. <$28 000 per year ------- <$540 / week
- b. $28000 - $52000 ------ $540 -$1000 / week
- c. $52000 - $83000----------$1000 - $1600 / week
- d. $83000-$125000-----------$1600 - $2400/week
- e. More than $125000--------> $2400/ week

224. **What is you approximate personal income before tax and other deductions?**

Mark only one oval.

- a. <$28 000 per year ------- <$540 / week
- b. $28000 - $52000 ------ $540 -$1000 / week
- c. $52000 - $83000----------$1000 - $1600 / week
- d. $83000-$125000-----------$1600 - $2400/week
- e. More than $125000--------> $2400/ week

Section 28 - Mental health - attitudes

225. **Mental health attitudes**

Mark only one oval per row.

	Yes	No	Unsure
a. Did the last person you had sex with know or did you tell them that you have mental health problems?	⬭	⬭	⬭
b. Did your regular sexual partner know or did you tell them that you have mental health problems?	⬭	⬭	⬭
c. Do you think your MH care team should have a role in your sexual health?	⬭	⬭	⬭

226. **Do you think you should be able to receive the following services at your mental health service?**

Check all that apply.

- ☐ Contraceptive advice
- ☐ Prescriptions for contraceptives
- ☐ Screening for STI's
- ☐ Treatment for STI's
- ☐ Advice about healthy sexual relationships
- ☐ Managing sexual dysfunction?
- ☐ Support around sexual identity
- ☐ Support for sexual abuse or coercion

Section 29 - Quality of life

227. **Do you think that sexual experiences influence your Quality of life?**

Mark only one oval.

- Yes
- No
- Maybe

228. **Do you think that improving your sexual function will improve your quality of life**

Mark only one oval.

- Yes
- No
- Maybe

Section 30 - Sexual Dysfunction Questionnaire

229. **Sexual dysfunction questionnaire**

Mark only one oval per row

	Always	Often	Sometimes	Rarely	Never
I am satisfied with my sex life					
I have sexual fantasies					
I have sexual dreams					
I like to talk about things that concern sexuality					
I like to tell jokes involving sex					
I feel uninhibited towards sexuality					
I like to keep sexuality hidden					
I speak about sexuality with my partner					
I live sexually in a rigid manner					
I would live better without sexuality					
My sex life is planned					
I avoid situations that arouse my sexuality					
Sexuality creates worry for me					
I like watching movies or scenes involving sex					
I like to talk during sex					
I reach orgasm during sex					
during sex I "let go"					
Sexuality scares me					
I like to have an active role in my sexuality					

Section 31 - Anti-psychotic and Sexual Functioning Questionnaire (ASFQ)

230. **Indication for use of current anti-psychotic?**

231. **List medications and dose (include non anti-psychotics) taken prior to current anti-psychotic?**

232. **How long did you use the previous anti-psychotic?**

Mark only one oval.

- a. No use of any other antipsychotic in the month before the current antipsychotic
- b. < 1week
- c. 1-2 weeks
- d. 2-6 weeks
- e. 6 weeks – 3 months
- f. > 3 months

233. **What was the main reason for quitting the previous anti-psychotic?**

234. **Current medication, anti-psychotic and co-medication, including dose?**

235. **Can you describe the experienced result(s) of treatment with the current anti-psychotic medication?**

Mark only one oval.

- a. Unknown
- b. Significant deterioration
- c. Mild deterioration
- d. Unchanged
- e. Mild improvement
- f. Significant improvement

236. **Which side effects did you experience from the anti-psychotic (side effects mentioned spontaneously)?**

237. **Have you noticed a change in sexual desire since using the current anti-psychotic?**

Mark only one oval.

- a. Unknown
- b. Significantly decreased
- c. Mildly decreased
- d. Unchanged
- e. Mildly increased
- f. Significantly increased

238. **Has your ability to achieve orgasm changed since using the current anti-psychotic?**

Mark only one oval.

- a. Unknown
- b. Significantly decreased
- c. Mildly decreased
- d. Unchanged
- e. Mildly increased
- f. Significantly increased

239. **Galactorrhea: In the past 4 to 6 weeks, did milk leak from your breasts / nipples?**

Mark only one oval.

- Yes
- No
- Unknown

240. **In the past 4 to 6 weeks, did you notice a swelling of your breasts / nipples?**

Mark only one oval.

- Yes
- No
- Unknown

241. **Have your ability to have an erection changed since using the current anti-psychotic?**

Mark only one oval.

- a. Unknown
- b. Significantly decreased
- c. Mildly decreased
- d. Unchanged
- e. Mildly increased
- f. Significantly increased
- g. Priapism

242. **Have you noticed a change in the volume of the ejaculate since using the current anti-psychotic?**

Mark only one oval.

- a. Unknown
- b. Significantly decreased
- c. Mildly decreased
- d. Unchanged
- e. Mildly increased
- f. Significantly increased
- g. Priapism

243. **Did you have sexual intercourse with a partner in the last 4 to 6 weeks?**

Mark only one oval.

- Yes
- No

Section 32 - Brief Psychiatric Rating Scale

The Brief Psychiatric Rating Scale (BPRS) is rating scale which a clinician or researcher may use to measure psychiatric symptoms such as depression, anxiety, hallucinations and unusual behaviour.[1] Each symptom is rated 1-7 and depending on the version between a total of 18-24 symptoms are scored

244. *Mark only one oval per row.*

	Not assessed	Not present	Very Mild	Mild	Moderate	Moderately Severe	Severe	Extremely Severe
Somatic concern								
2 Anxiety								
3 Depression								
4 Suicidality								
5 Guilt								
6 Hostility								
7 Elated Mood								
8 Grandiosity								
9 Suspiciousness								
10 Hallucinations								
11 Unusual thought content								
12 Bizarre behaviour								
13 Self-neglect								
14 Disorientation								
15 Conceptual disorganisation								
16 Blunted affect								
17 Emotional withdrawal								
18 Motor retardation								
19 Tension								
20 Uncooperativeness								
21 Excitement								
22 Distractibility								
23 Motor hyperactivity								
24 Mannerisms and posturing								

Section 33 MARS – Medication Adherence Rating Scale:

245. **Answer yes or no to the following**

Mark only one oval per row.

	Yes	No
Do you ever forget to take your medication?	⬭	⬭
Are you careless at times at taking your medication?	⬭	⬭
When you feel better do you sometimes stop taking your medication?	⬭	⬭
Sometimes if you feel worse when you take the medication, do you stop taking it?	⬭	⬭
I take my medication only when I am sick?	⬭	⬭
It is unnatural for my mind and body to be controlled by medication?	⬭	⬭
My thoughts are clear on medication	⬭	⬭
By staying on medication, I can prevent getting sick	⬭	⬭
I feel weird, like a zombie, on medication	⬭	⬭
The medication makes me feel tired and sluggish	⬭	⬭

Section 34 - Wrap up

246. **How embarrassing did you find the questionnaire?**

Mark only one oval.

	1	2	3	4	5	
Not at all	⬭	⬭	⬭	⬭	⬭	extremely

247. **On a scale of 1 to 10, how honest were you with your answers to the questions?**

Mark only one oval.

	0	1	2	3	4	5	6	7	8	9	10	
Not at all	⬭	⬭	⬭	⬭	⬭	⬭	⬭	⬭	⬭	⬭	⬭	Totally honest
